# Gestational and lactational dietary supplementation with live yeast partially attenuates inflammatory responses to lipopolysaccharide challenge in newly weaned piglets

**DOI:** 10.1093/jas/skaf435

**Published:** 2025-12-15

**Authors:** Yuechi Fu, Abiola S Lawal, Timothy A Johnson, Theresa M Casey, Jun Xie, Olayiwola Adeola, Kolapo M Ajuwon

**Affiliations:** Department of Animal Sciences, Purdue University, West Lafayette, IN 47907; Department of Animal Sciences, Purdue University, West Lafayette, IN 47907; Department of Animal Sciences, Purdue University, West Lafayette, IN 47907; Department of Animal Sciences, Purdue University, West Lafayette, IN 47907; Department of Statistics, Purdue University, West Lafayette, IN 47907; Department of Animal Sciences, Purdue University, West Lafayette, IN 47907; Department of Animal Sciences, Purdue University, West Lafayette, IN 47907

**Keywords:** inflammatory response, lipopolysaccharide, live yeast, piglet, sow

## Abstract

Weaning is an abrupt event in the life of piglets that adversely affects metabolic homeostasis, leading to poor nutrient absorption, increased susceptibility to enteric pathogens, and reduced growth performance. Few studies have examined the effects of maternal dietary live yeast (LY) supplementation on the responses of piglets subjected to an immunological challenge immediately after weaning. The aim of this study was to evaluate the effects of gestational and lactational dietary LY supplementation on inflammatory and antioxidant markers in newly weaned piglets challenged with lipopolysaccharide (LPS). On day 77 of gestation, 40 sows were randomly assigned to two dietary treatments: without (CON) or with LY supplementation at 0.05% of the diet during gestation and 0.1% during lactation. Within 24 h postweaning, 16 piglets with similar weights were selected from each maternal group and intraperitoneally injected with sterile saline or LPS, resulting in four treatment groups (*n *= 8): 1) CON + saline (CS), 2) LY + saline (YS), 3) CON+ LPS (CLPS), and 4) LY + LPS (YLPS). Rectal temperature was measured hourly for 4 h post-injection, after which piglets were euthanized. Samples of the mesenteric lymph node, liver, muscle, and intestinal mucosa were collected at 4 h post-injection to detect maternal LY-induced physiological changes in piglets. Results showed that YLPS piglets tended to have a lower rectal temperature than CLPS piglets at 3 h post-injection (*P *= 0.09). Levels of tumor necrosis factor (TNF)-α were decreased in the ileal mucosa of YLPS piglets compared with CLPS piglets (*P *< 0.05). Additionally, piglets from LY-supplemented sows had higher mRNA abundance of *interleukin* (*IL*)*-6*, *IL-10*, *TNF-α*, and *IL-1β* in the ileal mucosa, with higher protein abundance of E-cadherin in the jejunal mucosa than those from CON sows (*P *< 0.05). In the liver, YLPS piglets had lower mRNA abundance of *nuclear factor kappa B* (*NF-κB*) and *toll-like receptor 4* than CLPS piglets (*P *< 0.05). In the mesenteric lymph node, piglets from LY-supplemented sows had lower gene expression of *NF-κB* and *myeloid differentiation factor 88* than those from CON sows (*P *< 0.05). These results suggest that maternal dietary LY supplementation may confer protective effects against bacterial endotoxin exposure by attenuating inflammatory responses in newly weaned piglets, with implications for improved resilience to certain gram-negative bacterial infections, such as *Escherichia coli*, after weaning.

## Teaser

Gestational and lactational dietary supplementation with live yeast partially attenuates lipopolysaccharide-induced inflammation in offspring.

## Introduction

Weaning is a routine procedure in pig husbandry, although it is associated with nutritional, immunological, and physiological disturbances in piglets (Pluske et al., 2018). These perturbations result from multiple events that occur during weaning, including transportation, handling, sudden dietary transition, and environmental change. Consequently, weaning stress-induced diarrhea is associated with gut dysbiosis, growth retardation, and increased mortality (Tang et al., 2022). As this period is associated with poor pig performance, several management, dietary, and feeding strategies have been employed to mitigate the adverse effects of weaning stress. Some of these strategies target the gestating and lactating sows, with the expectation that the benefits are transferable to piglets after weaning. A few of these approaches include provision of adequate housing during gestation, adjusting sow feeding or supplementing sow diets with probiotics and prebiotics during pregnancy and lactation (Blavi et al., 2021).

Yeast and yeast-based byproducts, including yeast culture, live yeast (LY), and yeast cell wall components such as β-glucans and mannan oligosaccharides, have been utilized as dietary supplements to modulate metabolic processes and enhance immune function in food animal production across various growth stages (Burdick Sanchez et al., 2021; Patterson et al., 2022). Commercially available LY products for animal feeding are typically manufactured in coated or encapsulated granular forms to protect yeast stability and viability during diverse storage and environmental conditions (Arslan et al., 2015). Live yeast exerts probiotic effects through viable yeast cells and prebiotic functions through polysaccharide-rich cell wall fractions and fermentation-derived metabolites, which support gastrointestinal health and host physiological function through multiple mechanisms (Fan et al., 2025). These include modulation of the immune system by promoting immune cell proliferation or activating macrophages and neutrophils to release inflammatory cytokines (Kogan and Kocher, 2007; Monroy-Salazar et al., 2012), enhancement of the host antioxidant defense system by upregulating the synthesis of antioxidant enzymes such as superoxide dismutase and catalase (Long et al., 2021), or supporting intestinal barrier function by stimulating mucin secretion and increasing expression of key junctional proteins, including proteins located in tight junctions and adherens junctions (Fan et al., 2024). Moreover, when fermented, LY cell wall components such as β-glucan and mannan oligosaccharides produce short-chain fatty acids and contribute to gastrointestinal acidification, thereby inhibiting pathogenic bacteria, reshaping gut microbiota composition, and increasing microbial diversity (Kiros et al., 2019). Consequently, research evidence indicates that supplementing LY, either directly in nursery pig diets or indirectly through the sow, enhances piglet growth performance, nutrient digestibility (Lu et al., 2019), and intestinal development (Fu et al., 2024), while also reducing the instances of antimicrobial resistance to antibiotics (Chance et al., 2022).

Exposure to bacterial pathogens represents a major challenge for weanling pigs. This period represents a time during which piglets are vulnerable to intestinal and systemic inflammation in response to bacterial infection from organisms such as *Escherichia coli*. Lipopolysaccharide (LPS) obtained from *E. coli* is often used to model this inflammatory response (Yao et al., 2017; Guevara et al., 2024). Although previous studies have reported the effects of maternal or postweaning LY supplementation on intestinal health and immune function in pigs several weeks after weaning (Trckova et al., 2014; Law et al., 2024), few have investigated the effects of maternal LY supplementation on tissue-specific inflammatory responses in piglets facing an inflammatory stimulus immediately after weaning. Therefore, the objective of the present study was to determine the impacts of supplementing LY to sow diets on inflammatory and antioxidant markers in selected tissues (intestinal mucosa, liver, muscle, and mesenteric lymph node) of piglets challenged with LPS immediately after weaning.

## Materials and Methods

All protocols used in this study were approved by the Purdue University Institutional Animal Care and Use Committee (No. 1111000145).

### Animals and experimental design

The management and dietary treatment of sows during late gestation and lactation were described in our previous study (Fu et al., 2024). Briefly, on day 77 of gestation, a total of forty sows (Landrace × either Duroc or Yorkshire) were randomly assigned to two dietary groups based on their expected farrowing date and parity (*n *= 20): basal diet only (CON) or the basal diet supplemented with live yeast (LY, *Saccharomyces cerevisiae*, Vistacell, AB Vista, Marlborough, Wiltshire, UK). The LY product was mixed directly into the feed at inclusion rates of 0.05% during gestation and 0.10% during lactation. According to manufacturer specifications and previous studies using this product (Lu et al., 2019), LY contains approximately 2 × 10^10^ CFU/g viable yeast cells; however, viable counts were not quantified in the current study. The basal diet (Table 1) was formulated to meet or exceed the nutrient requirements for primiparous sows according to the National Research Council (NRC, 2012).

**Table 1.** Ingredient composition and nutrient levels of gestation and lactation diets (%, as-fed basis^1^)

Ingredient, %	Gestation	Lactation
**Corn**	76.95	57.62
**SBM, 47.5% CP**	17.50	35.00
**Swine Grease**	1.20	3.00
**Limestone**	1.69	1.63
**Monocalcium phosphate**	1.30	1.50
**Sow vitamin premix^2^**	0.15	0.15
**Choline Chloride (60%)**	0.10	0.10
**Rovimix-CarniChrom^3^**	0.01	0.01
**Phytase^4^**	0.10	0.10
**NaCl**	0.50	0.50
**Clarify^5^**	0.21	0.10
**Defusion Plus^6^**	0.25	0.25
**Availa Zn^7^**	0.04	0.04
**Total**	100.00	100.00
***Calculated composition***		
** ME, kcal/kg**	3286.3	3352.8
**CP, g/kg**	146.9	214.5
**Ca, g/kg**	9.0	9.6
**Total P, g/kg**	5.8	7.2
**STTD^8^ P, g/kg**	3.7	4.5
**SID Lys^9^, g/kg**	6.0	10.3

1 Live yeast (*Saccharomyces cerevisiae*, 2 × 10^10^ CFU/g, Vista Cell, AB Vista, Marlborough, Wiltshire, UK) was added to the control diets as a replacement for corn in a premix to supply 0.05% and 0.1% for gestation and lactation, respectively.

2 Sow vitamin premix, Provimi, Lewisburg, OH. Provided per kg of diet: vitamin A, 11,161 IU; vitamin D_3_, 2,545 IU; vitamin E, 66 IU; vitamin K, 1.42 mg; riboflavin, 6.6 mg; pantothenic acid, 23.6 mg; niacin, 44.2 mg; B_12_, 31 µg; biotin, 0.44 mg; folic acid, 1.62 mg; thiamine, 0.25 mg; pyrdoxine-B_6_, 0.25 mg; iron, 129 mg; zinc, 125 mg; manganese, 60 mg; copper, 20 mg; iodine, 1.26 mg; selenium, 0.3 mg; cobalt, 0.02 mg; calcium, 950 mg; sodium, 800 mg, chloride, 1200 mg; phytase, 371 FTU; and estimated 0.12% phosphorus release from the phytase.

3 Rovimix-CarniChrom, DSM Nutritional Products, NJ. Provided chromium at 0.20 mg/kg of diet.

4 Phytase (Quantum Blue, AB Vista, Marlborough, UK) was included to provide 500 phytase units (FTU)/kg of diet. No matrix values were applied during diet formulation.

5 Clarifly Larvicide 0.67%, Central Life Sciences, Schaumberg, IL.

6 Defusion Plus preservatives, Provimi, Lewisburg, OH.

7 Availa Zn 120, Zinpro, Eden Prairie, MN.

8 Standardized total tract digestible.

9 Standardized ileal digestible lysine.

### *E. coli* lipopolysaccharide challenge

**Figure 1.**
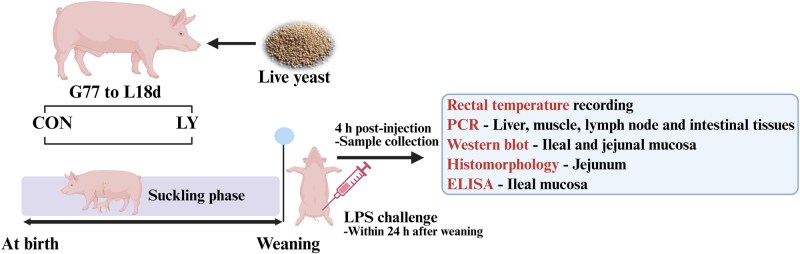
Schematic view of the experimental design (Created in https://BioRender.com). Abbreviations: CON, control; G, gestation; L, lactation; LY, live yeast; LPS, lipopolysaccharide.

Within 24 h postweaning, thirty-two healthy male piglets from sixteen sows per treatment (one piglet per sow, average weight of the CON group: 6.11 ± 0.20 kg, average weight of the LY group: 6.23 ± 0.23 kg) were selected on the day of weaning (average weaning age: 19.2 days), and the weaned piglets were individually housed in a separate cage (0.9 × 0.4 × 0.6 m) prior to the immunological challenge. Lipopolysaccharide (LPS; *E. coli* serotype O55: B5; Sigma Chemical Inc., St Louis, MO, USA) was reconstituted in sterile saline (9 g/L NaCl) to prepare a solution at a concentration of 2.5 mg/L. Piglets in each treatment were intraperitoneally injected with 25 μg LPS per kg BW to induce an inflammatory response (Weber and Kerr, 2008; Gu et al., 2017), while the other sixteen were intraperitoneally injected with an equivalent amount of sterile saline. This resulted in four treatment groups (*n *= 8 per group): 1) control + saline (CS), 2) live yeast + saline (YS), 3) control + LPS (CLPS), and 4) live yeast + LPS (YLPS). The piglets were euthanized 4 h after lipopolysaccharide challenge (Gu et al., 2017). A schematic view of the experimental design is provided in Figure 1.

### Sample collection

Rectal temperature was measured using a digital thermometer before the LPS injection for baseline measurement and then hourly thereafter. At 4 h post-injection, piglets were euthanized for collecting mesenteric lymph node, liver, muscle, jejunal mucosa, and ileal mucosa. Mucosal samples were collected from gut sections corresponding to the jejunum and ileum by scraping the lining of the gastrointestinal tract using a glass slide. Mucosal scrapings were transferred into microfuge tubes, one tube contained TRIzol reagent (Invitrogen, Carlsbad, CA, USA) to preserve for RNA extraction, and the other tube stored samples for protein extraction, both samples were snap-frozen in liquid nitrogen and stored at −80°C until analysis. Additionally, a 5 cm segment of the mid jejunum was flushed with ice-cold phosphate-buffered saline and placed in 10% neutral-buffered formalin for histological analysis.

### Jejunal histomorphology

Formalin-fixed jejunal samples were processed at the Purdue Histology and Phenotyping Laboratory (Purdue University, West Lafayette, IN, USA) for histology analysis. Six tissue sections containing intact lamina propria were selected from each piglet for measurement. Villus height (VH) and crypt depth (CD) were measured in micrometers using ImageJ software (NIH, Bethesda, MD, USA). For each sample, VH and CD values were averaged, and the villus height-to-crypt depth (VH:CD) ratio was calculated.

### RT-qPCR

Gene expression in tissue samples was measured using a reverse transcription quantitative polymerase chain reaction (RT-qPCR), as previously described (Fu et al., 2024). Briefly, total RNA was extracted from frozen tissue samples including liver, muscle, lymph node, as well as the mucosa of jejunum and ileum using TRIzol reagent (Invitrogen, Carlsbad, CA, USA). The concentration of RNA was quantified using a Nanodrop 1000 instrument (Thermo Scientific, Waltham, MA, USA), and the RNA integrity was verified using 1% agarose gel electrophoresis. One μg of RNA was used for reverse transcription using M-MLV reverse transcriptase (Promega, Madison, WI, USA). Afterward, transcript amplification was performed using the CFX-96 real-time PCR detection system (Bio-Rad, Hercules, CA, USA) with the SYBR green RT-PCR mix (Bimake, Houston, TX, USA). The PCR protocol was as follows: 1 cycle of 95°C for 3 min and 40 cycles of 95°C for 10 s, followed by 30 s at an annealing temperature for each primer and 65°C for 30 s. Inflammatory and toll-like receptor 4 pathway-related markers, including *interleukin* (*IL*)*-6, IL-1β, IL-8, IL-10, tumor necrosis factor* (*TNF*)*-α*, *toll-like receptor 4* (*TLR4*), *inhibitor of kappa B alpha* (*IKBA*), *myeloid differentiation factor 88* (*MYD88*), *nuclear factor kappa B* (*NF-κB*), and antioxidant markers, *superoxide dismutase* (*SOD*)*1*, *catalase* (*CAT*), and *glutathione peroxidase* (*GPX*)*1* were detected by RT-qPCR using their respective primers. Target mRNA levels were normalized to *Glyceraldehyde 3-phosphate dehydrogenase* (*GAPDH*) and analyzed using the 2^−ΔΔCt^ method, with results expressed as fold changes relative to the CS group (Livak and Schmittgen, 2001). The primer sequences are provided in Supplementary Table S1.

### ELISA

Total protein levels in the ileal mucosal homogenates were determined using a Pierce BCA protein assay (Thermo Scientific, Waltham, MA, USA). Concentrations of TNF-α and IL-10 were quantified using respective commercial ELISA kits TNF-α and IL-10 (Life Diagnostics, Inc., West Chester, PA, USA) following the manufacturer’s protocols. Data were expressed as pg/mg of protein.

### Western blotting

The methods for analyzing tight junction proteins by Western blot were described in Fu et al. (2024). Briefly, ileal and jejunal mucosa samples were homogenized and lysed in 1 × radio-immunoprecipitation assay buffer supplemented with 1% phosphatase and protease inhibitor cocktail (Thermofisher, Waltham, MA, USA) using TissueLyser (Qiagen, Valencia, CA, USA) at 30 Hz twice for 2 min. Protein concentrations were determined by a bicinchoninic acid assay kit (Sigma-Aldrich, St Louis, MO, USA). Proteins were resolved on a 10% SDS polyacrylamide gel and transferred onto nitrocellulose membranes (Bio-Rad, Hercules, CA, USA) using a semi-dry transfer system (Bio-Rad, Hercules, CA, USA). After transfer, membranes were cut into sections according to molecular weight from the marker lane and blocked with 5% bovine serum albumin dissolved in Tris-buffered saline with 0.1% Tween at room temperature for 2 h, and then incubated with corresponding primary antibodies at a dilution of 1:1,000 overnight at 4°C. The following primary antibodies were used: anti-claudin-4 (Catalog No. 329400; Life Technologies, Carlsbad, CA, USA), anti-claudin-3, occludin (Catalog No. ab15102 and ab31721, respectively; Abcam, Cambridge, MA, USA), anti-β-Actin (13E5) rabbit mAb, and E-cadherin (#4970 and #3195; Cell Signaling Technology, MA, USA). Membranes were washed at least four times with TBST and then incubated with corresponding secondary antibodies (anti-rabbit or anti-mouse IgG-horseradish peroxidase; Cell Signaling Technology, Danvers, MA, USA) at a dilution of 1:25,000 at room temperature for 1 h. Protein bands were visualized on a FluorChem imager (Proteinsimple, San Jose, CA, USA) using immobilon chemiluminescent HRP substrate (Millipore, Billerica, MA, USA). Raw densitometric readings were obtained and quantified using ImageJ (NIH, Bethesda, MD, USA) and averaged for eight samples from each treatment. The relative abundance of each target protein was expressed as the ratio of target protein to β-actin.

### Statistical analysis

Data was analyzed by two-way analysis of variance using the PROC MIXED procedure of SAS (SAS Inst. Inc., Cary, NC, USA) for a split-plot design, with sow diet as the whole-plot and immunological challenge as the sub-plot. The Shapiro-Wilk test was used to analyze the normality of the data. Significance was set at *P *≤ 0.05, and a tendency was defined at 0.05 < *P *≤ 0.10. Results were illustrated using GraphPad Prism 7.03 (GraphPad Software Inc., San Diego, CA, USA).

## Results

### Effects of maternal dietary live yeast supplementation on rectal temperature

**Figure 2.**
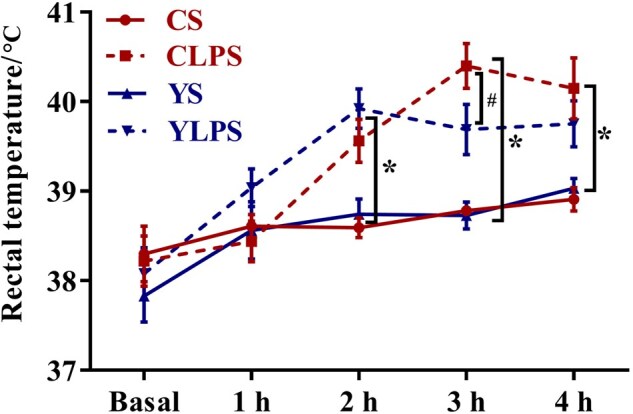
Effects of maternal dietary live yeast supplementation on rectal temperature of newly weaned piglets in response to a lipopolysaccharide challenge (*n *= 8). Abbreviations: CS, control + saline; YS, yeast + saline; CLPS, control + lipopolysaccharide; YLPS, yeast + lipopolysaccharide. *Indicates significances (*P *≤ 0.05); ^#^Represents trends (0.05 < *P *≤ 0.10).

At 2 h post-injection, LPS challenge led to increased rectal temperature compared with saline group, and this effect was still observable up to 4 h post-injection (*P *< 0.05, Figure 2). At 3 h post-injection, piglets in the YLPS group tended to have a lower rectal temperature compared to those in the CLPS group (*P *= 0.09).

### Effects of maternal dietary live yeast supplementation on expression of inflammatory- and antioxidant-related markers in the intestinal mucosa

**Figure 3.**
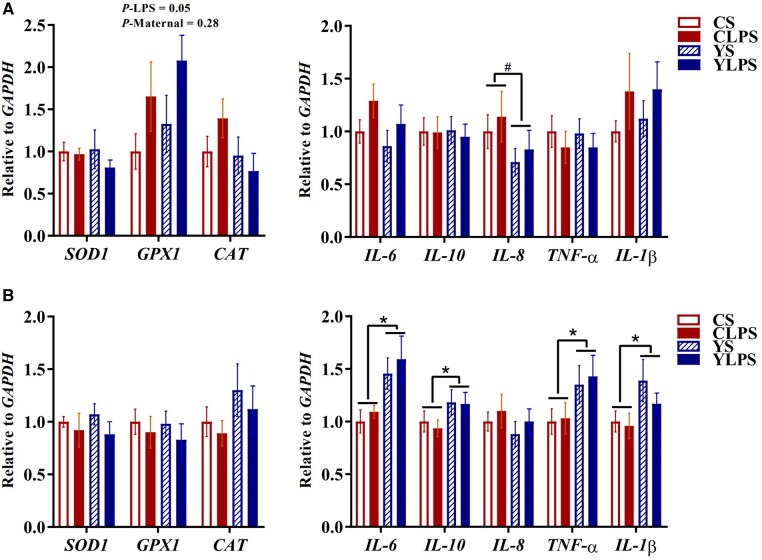
Effects of maternal dietary live yeast supplementation on mRNA abundance of inflammatory- and antioxidant-related markers in the (A) jejunal and (B) ileal mucosa of newly weaned piglets in response to a lipopolysaccharide challenge (*n *= 8). ^*^Indicates significances (*P *≤ 0.05); ^#^Represents trends (0.05 < *P *≤ 0.10). Abbreviations: CAT, catalase; CS, control + saline; CLPS, control + lipopolysaccharide; GAPDH, glyceraldehyde 3-phosphate dehydrogenase; GPX1, Glutathione peroxidase 1; IL, interleukin; SOD, superoxide dismutase; TNF, tumor necrosis factor; YS, yeast + saline; YLPS, yeast + lipopolysaccharide.

In the jejunal mucosa, LPS challenge increased mRNA abundance of *GPX1* compared with the saline group (*P *= 0.05, Figure 3A), whereas mRNA abundance of *SOD1* and *CAT* was unaffected (*P *> 0.05). In addition, piglets from LY-supplemented sows tended to have lower mRNA levels of *IL-8* (*P *= 0.09), while neither maternal diet nor LPS challenge influenced mRNA abundance of inflammatory cytokines, including *IL-6*, *IL-10*, *TNF-α*, and *IL-1β* (*P *> 0.05).

In the ileal mucosa, piglets from LY-supplemented sows had higher mRNA abundance of *IL-6*, *IL-10*, *TNF-α*, and *IL-1β* (*P *< 0.05, Figure 3B), whereas no effect of maternal diet was observed for mRNA abundance of *IL-8* or for LPS-induced effects in these genes (*P *> 0.05). Expression of antioxidant-related genes, including *SOD1*, *GPX1*, and *CAT* in the ileal mucosa did not differ in response to maternal diet or LPS challenge (*P *> 0.05). Additionally, TNF-α concentrations in the ileal mucosa were higher in piglets from CON sows than those from LY-supplemented sows (*P *< 0.05; Table 2), whereas no effect of LPS challenge was detected (*P *> 0.05). An interaction between maternal diet and LPS challenge was observed for both TNF-α and IL-10 concentrations (*P *= 0.05).

**Table 2.** Effects of maternal dietary live yeast supplementation on IL-10 and TNF-α concentrations in the ileal mucosa of newly weaned piglets following a lipopolysaccharide challenge

Item (pg/mg)	CON		LY		*SEM*	*P*-value		
	Saline	LPS	Saline	LPS		Maternal diet	Challenge	Maternal diet × Challenge
**IL-10**	13.99	16.26	19.31	12.96	2.03	0.62	0.33	0.05
**TNF-α**	108.14^ab^	120.39^a^	106.40^ab^	90.43^b^	7.07	0.03	0.78	0.05

a-b indicates significances (*P *≤ 0.05). Abbreviations: CON, control; LY, live yeast; IL, interleukin; TNF, tumor necrosis factor; (*n *= 8).

### Effects of maternal dietary live yeast supplementation on the expression of inflammatory- and antioxidant-related markers in the liver and muscle

In the liver, mRNA abundance of *CAT* was downregulated by LPS (*P *< 0.05, Table 3), while no maternal dietary effect was observed (*P *> 0.05). Neither maternal diet nor LPS challenge influenced mRNA abundance of *SOD1* and *GPX1* (*P *> 0.05). The mRNA abundance of both pro- and anti-inflammatory cytokines, including *TNF-α*, *IL-6*, and *IL-10*, was upregulated in the LPS-challenged groups (*P *< 0.05), but maternal diet had no effects on these genes (*P *> 0.05). LPS challenge also elevated the mRNA abundance of *TLR4*, *MYD88*, *IKBA*, and *NF-κB* (*P *< 0.05), while effects of maternal diet were detected for *NF-κB* (*P *< 0.05) and a tendency was observed for *TLR4* (*P *= 0.07), with piglets from LY-supplemented sows exhibiting lower mRNA abundance under LPS challenge compared with those from CON sows. In addition, interactions were observed for mRNA abundance of *TLR4* and *NF-κB* (*P *< 0.05).

**Table 3.** Effects of maternal live yeast supplementation on mRNA abundance of inflammatory- and antioxidant-related markers in the liver and lymph node of newly weaned piglets in response to a lipopolysaccharide challenge

Maternal diet	CON		LY		*SEM*	*P*-value		
	Saline	LPS	Saline	LPS		Maternal diet	Challenge	Maternal diet × Challenge
**Liver**								
*** SOD1***	1.00	0.92	1.37	1.02	0.30	0.41	0.45	0.63
*** GPX1***	1.00	1.45	1.08	1.49	0.31	0.84	0.16	0.94
***CAT***	1.00	0.61	1.30	0.67	0.12	0.17	0.0007	0.34
*** IL-6***	1.00	2.85	0.87	2.72	0.24	0.55	<0.0001	0.98
*** IL-10***	1.00	1.70	0.91	1.55	0.16	0.45	0.0005	0.84
*** TNF-α***	1.00	1.57	1.19	1.72	0.17	0.31	0.003	0.92
***TLR4***	1.00^c^	2.91^a^	1.09^c^	1.57^bc^	0.33	0.07	0.002	0.04
***IKBA***	1.00	2.14	1.06	3.32	0.47	0.84	<0.0001	0.63
***MyD88***	1.00	3.83	1.03	3.31	0.41	0.54	<0.0001	0.49
***NF-κB***	1.00^c^	5.39^a^	1.02^c^	3.27^b^	0.42	0.02	<0.0001	0.02
**Lymph node**								
*** SOD1***	1.00	0.53	0.78	0.52	0.12	0.31	0.004	0.36
***GPX1***	1.00	0.95	0.64	0.78	0.16	0.13	0.78	0.56
*** CAT***	1.00	0.74	0.65	0.52	0.13	0.04	0.15	0.59
***IL-6***	1.00	1.22	1.07	1.39	0.20	0.53	0.16	0.81
*** IL-10***	1.00^b^	2.01^a^	1.03^b^	1.15^b^	0.17	0.03	0.004	0.02
*** TNF-α***	1.00	1.64	1.30	1.16	0.23	0.70	0.27	0.10
*** TLR4***	1.00	1.83	1.13	1.41	0.23	0.49	0.01	0.20
*** IKBA***	1.00	0.76	0.54	0.19	0.19	0.19	0.85	0.30
*** MyD88***	1.00	1.79	0.78	0.92	0.21	0.02	0.04	0.13
***NF-κB***	1.00	0.96	0.64	0.65	0.15	0.02	0.93	0.84

a-c indicates significances (*P *≤ 0.05). Abbreviations: CAT, catalase; CON, control; GAPDH, glyceraldehyde 3-phosphate dehydrogenase; GPX1, glutathione peroxidase 1; IKBA, inhibitor of nuclear factor kappa B Alpha; IL, interleukin; LY, live yeast; MYD88, myeloid differentiation factor 88; NF-κB, nuclear Factor Kappa B; SOD, superoxide dismutase; TNF, tumor necrosis factor; TLR, toll-like receptor; (*n *= 8).

In the muscle, a trend toward an interaction between maternal diet and LPS challenge was observed for mRNA abundance of *GPX1* (*P *= 0.10; Supplementary Table S2), along with a tendency for higher mRNA abundance of *IL-6* in LPS-challenged piglets (*P *= 0.09). However, neither maternal diet nor LPS challenge affected mRNA abundance of *SOD1*, *CAT*, *IL-10*, or *TNF-α* (*P *> 0.05).

### Effects of maternal dietary live yeast supplementation on expression of inflammatory- and antioxidant-related markers in the lymph node

In the lymph node, LPS challenge reduced mRNA abundance of *SOD1* compared with the saline group (*P *< 0.05, Table 3), while no effect of maternal diet was observed (*P *> 0.05). Neither maternal diet nor LPS challenge affected *GPX1* expression (*P *> 0.05). Piglets from LY-supplemented sows had lower gene expression of *CAT* than those from CON sows (*P *< 0.05), while no effect of LPS challenge was detected (*P *> 0.05). In response to LPS challenge, piglets from CON sows showed higher mRNA abundance of *IL-10* than those from LY-supplemented sows (*P *< 0.05), with an interaction between maternal diet and LPS challenge observed for *IL-10* (*P *< 0.05). Neither maternal diet nor LPS challenge affected mRNA abundance of *IL-6* (*P *> 0.05), whereas a trend toward an interaction between maternal diet and LPS challenge was observed for mRNA abundance of *TNF-α* (*P *= 0.10). In addition, LPS challenge upregulated mRNA abundance of *TLR4* and *MYD88* compared with the saline group (*P *< 0.05), while no effects of maternal diet were detected (*P *> 0.05). Furthermore, piglets from LY-supplemented sows had lower mRNA abundance of *NF-κB* and *MYD88* than those from CON sows (*P *< 0.05); however, neither maternal diet nor LPS challenge affected mRNA abundance of *IKBA* (*P *> 0.05).

### Effects of maternal dietary live yeast supplementation on jejunal histomorphology and tight junction protein expression in the intestinal mucosa

**Figure 4.**
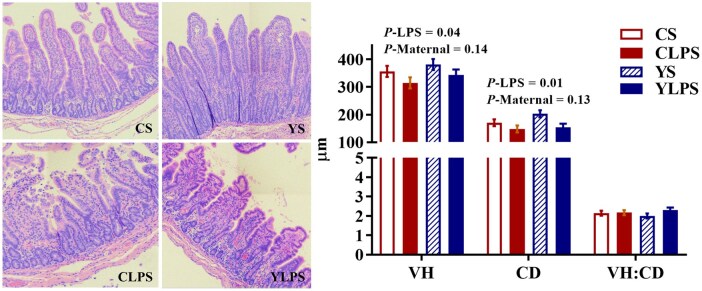
Effects of maternal dietary live yeast supplementation on jejunal histomorphology of newly weaned piglets in response to a lipopolysaccharide challenge (*n *= 8). Left panel: representative image from 1 piglet per treatment; Right panel: quantification of 8 samples per treatment. Abbreviations: CD, crypt depth; CS, control + saline; YS, yeast + saline; CLPS, control + lipopolysaccharide; YLPS, yeast + lipopolysaccharide; VH, villus height; VH: CD, villus height-to-crypt depth ratio. Magnification: × 10.

**Figure 5.**
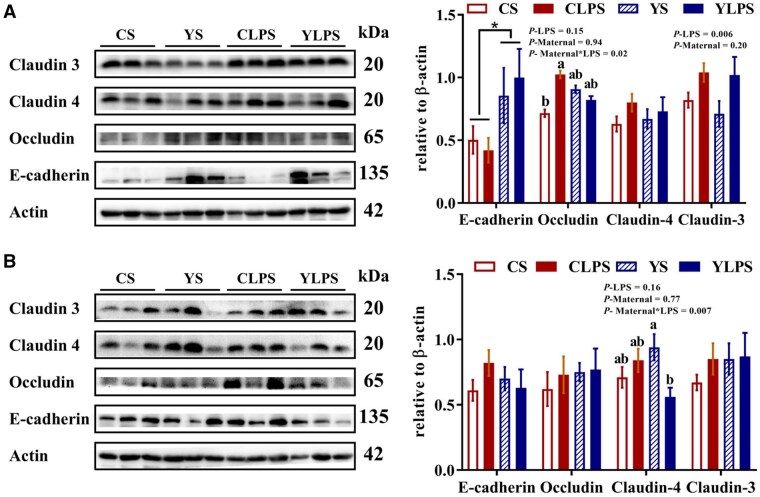
Effects of maternal dietary live yeast supplementation on tight junction protein expression in the (A) jejunal and (B) ileal mucosa of newly weaned piglets in response to a lipopolysaccharide challenge (*n *= 8). ^a-b^Least squares means without a common superscript differ at *P *< 0.05. ^*^Indicates effect of maternal diet (*P *< 0.05). Left panel: representative western blot; Right panel: quantification of 8 samples per treatment. Abbreviations: CS, control + saline; YS, yeast + saline; CLPS, control + lipopolysaccharide; YLPS, yeast + lipopolysaccharide.

Jejunal villus height and crypt depth were reduced in LPS-challenged piglets compared with the saline group (*P* < 0.05, Figure 4), whereas no effect of maternal diet was observed (*P *> 0.05). Neither maternal diet nor LPS challenge affected the villus-to-crypt ratio (*P *> 0.05). Piglets from LY-supplemented sows exhibited higher E-cadherin protein abundance in the jejunal mucosa compared with those from CON sows (*P *< 0.05; Figure 5A and Supplementary Figure S1—see online supplementary material for a colour version of this figure), while no maternal dietary effects were detected for protein abundance of occludin, claudin-3, and claudin-4 (*P *> 0.05). An interaction between maternal diet and LPS challenge was observed for occludin abundance (*P *< 0.05), and LPS challenge increased claudin-3 protein abundance in the jejunal mucosa (*P *< 0.05). In the ileal mucosa, an interaction between maternal diet and LPS challenge was observed for claudin-4 protein abundance (*P *< 0.05, Figure 5B and Supplementary Figure S1—see online supplementary material for a colour version of this figure), whereas neither maternal diet nor LPS challenge affected the protein abundance of E-cadherin, occludin, claudin-3, and claudin-4 (*P *> 0.05).

## Discussion

Maternal transmission of immunomodulatory components occurs both prenatally and postnatally. During gestation, inflammatory mediators, microbial metabolites, and other bioactive compounds can cross the placenta, whereas postnatally, cytokines, immunoglobulins, and growth factors are delivered through colostrum and milk, thereby shaping neonatal immune function and early growth (Gormley et al., 2024). Therefore, dietary intervention during pregnancy and lactation represents a strategic approach for modulating intestinal development, immune resilience, and antioxidant defenses in the offspring. In the present study, we investigated the effects of dietary live yeast (LY) supplementation to sows during late pregnancy and lactation on inflammatory and antioxidant markers in various tissues of newly weaned piglets subjected to a LPS challenge.

The LPS challenge led to elevated rectal temperature at 2 h post-injection and lasted until 4 h, confirming the establishment of an acute inflammatory response. This is in agreement with previous studies that reported LPS-induced hyperthermia in piglets (Carroll et al., 2004). At 3 h post-injection, piglets from LY-supplemented sows (YLPS) exhibited a trend toward lower rectal temperatures compared with piglets from CON sows (CLPS), suggesting that maternal dietary LY supplementation might attenuate the LPS-induced febrile response. Intestinal barrier dysfunction contributes to inflammation and oxidative stress by allowing the translocation of luminal components, such as LPS, bacteria, and antigens, into systemic circulation (Ghosh et al., 2020). This translocation activates immune response, leading to release of pro-inflammatory cytokines both locally and systemically (Kim et al., 2012). Although the LPS challenge had limited effects on local inflammatory responses in the jejunal and ileal mucosa, including the mRNA abundance of *IL-6*, *IL-10*, *TNF-α*, and *IL-1β*, piglets from LY-supplemented sows exhibited higher ileal mucosal mRNA levels of both pro- and anti-inflammatory cytokines (*IL-6*, *TNF-α*, *IL-1β*, and *IL-10*). The increased expression of these cytokines may be attributed to the immune-priming properties of β-glucans (a key component of the LY cell wall), which may have altered the immune properties of colostrum or milk during the suckling phase (Broadway et al., 2015). Moreover, a tendency for lower mRNA level of *IL-8* was observed in the jejunal mucosa of piglets from LY-supplemented sows. IL-8 is a chemokine primarily involved in the recruitment, activation, and migration of neutrophils to sites of mucosal injury or infection (Matsushima et al., 2022). The reduced *IL-8* expression in piglets from LY-supplemented sows suggests a reduced pro-inflammatory response in the gut mucosa of the pigs (Kaiserman et al., 2022). In agreement with this, these piglets also exhibited lower TNF-α concentrations in the ileal mucosa and a higher E-cadherin protein expression in the jejunal mucosa compared with piglets from CON sows. E-cadherin is a marker of cell-cell adhesion, and higher mucosal E-cadherin abundance may indicate a stronger mucosal barrier capable of limiting neutrophil infiltration during inflammatory response (Lialios and Alimperti, 2025). Therefore, maternal immunomodulatory benefits conferred by LY supplementation, possibly through alterations in the immune properties of colostrum and milk, may help suppress excessive mucosal inflammation in piglets (Lewicka et al., 2019). Although maternal systemic and metabolic biomarkers were not measured in the present study, previous studies have reported that maternal dietary supplementation with LY increases plasma immunoglobulin G and decreases liver-associated enzyme activities such as aspartate aminotransferase and gamma-glutamyl transpeptidase in sows (Peng et al., 2020), which may reflect an improved immunometabolic status in LY-supplemented sows. These maternal alterations, together with LY-induced changes in the immunological properties of colostrum and milk, may modify the immune environment transmitted to offspring both in utero and postnatally, through colostrum and milk, thereby contributing to the attenuated mucosal cytokine responses observed in YLPS piglets.

The liver serves as a central organ for metabolism and detoxification, responsible for processing toxins, purifying the blood, and eliminating waste products generated by other tissues (Zhou et al., 2022). Under normal living conditions, liver-resident macrophages in piglets are capable of clearing low levels of circulating LPS from the bloodstream (Guo et al., 2025). However, accumulation of LPS beyond the capacity of the liver causes hepatocyte damage and activates hepatic macrophages, triggering the release of inflammatory cytokines and the production of reactive oxygen species (Kaur et al., 2006). In the current study, LPS reduced mRNA abundance of antioxidant enzyme catalase (*CAT*) and upregulated inflammatory cytokines including *IL-6*, *IL-10*, and *TNF-α* in the liver, which is consistent with previous studies showing that LPS reduces antioxidant capacity and promotes the release of inflammatory cytokines (Wen et al., 2023; Yu et al., 2023). Moreover, LPS activates toll-like receptor 4 (TLR4) signaling in both hepatocytes and Kupffer cells, initiating a cascade of intracellular events central to innate immune activation (Nakamoto and Kanai, 2014). Upon LPS binding, TLR4 recruits the adaptor protein myeloid differentiation factor 88 (MYD88), which activates downstream kinases that phosphorylate the inhibitor of NF-κB, IKBA. Phosphorylated IKBA is subsequently ubiquitinated and degraded, thereby allowing NF-κB to translocate from the cytoplasm to the nucleus and induce transcription of pro-inflammatory cytokines such as TNF-α and IL-6 (Pålsson-McDermott and O’Neill, 2004). In the present study, LPS challenge activated markers of the TLR4-MYD88-NF-κB signaling pathway in piglets, as evidenced by increased the hepatic mRNA abundance of *TLR4*, *MYD88*, *NF-κB*, *IL-6*, and *TNF-α*. We also observed an increase in *IKBA* mRNA abundance, which likely reflects the NF-κB-mediated negative feedback commonly observed during inflammatory activation (Hoffmann et al., 2002). Notably, YLPS piglets exhibited lower hepatic *NF-κB* and *TLR4* mRNA abundance compared with CLPS piglets, suggesting a potential attenuation of innate immune activation. This modulation may be partially attributable to yeast-derived nucleotides. For instance, Li et al. (2022a) reported that maternal nucleotide supplementation inhibited TLR4 and NF-κB expression in LPS-treated piglets. However, the specific anti-inflammatory components of LY remain to be identified.

Lymph nodes are essential peripheral immune organs in mammals, characterized by a highly organized structure that facilitates migration and activation of immune cells, and functions as immunological checkpoints in host defense (Zhang et al., 2023). Despite their importance, limited studies have examined the immune response in lymph nodes, particularly in livestock species such as pigs. In the present study, LPS reduced the mRNA abundance of *SOD1* in the lymph node, a key antioxidant enzyme that mitigates oxidative damage by catalyzing the dismutation of superoxide radicals (Eleutherio et al., 2021). This reduction suggests that LPS-induced inflammation may be accompanied by compromised antioxidant defense mechanisms in both hepatic and lymphatic tissues. In addition, the LPS challenge increased the mRNA abundance of markers in the TLR4-mediated inflammatory pathway, including *TLR4* and *MYD88* in challenged piglets. It also elevated *IL-10* expression, reflecting a compensatory anti-inflammatory response (Alexander et al., 2021). However, maternal dietary LY supplementation reduced mRNA abundance of *MYD88* and *NF-κB* in the lymph nodes of piglets. Although NF-κB activation is primarily regulated post-translationally (Hayden and Ghosh, 2012), reductions in *NF-κB* and *MYD88* transcription may reflect a lower signaling capacity of the pathway to initiate inflammatory responses. Furthermore, the observed interaction for IL-10 between maternal diet and LPS challenge suggests that maternal LY modulates the piglet’s regulatory response to LPS. Rather than indicating immunosuppression, this reduced activation may reflect a more balanced inflammatory response, whereby maternal LY supplementation enhances tolerance and prevents excessive inflammatory response to LPS (Liu et al., 2023). Our recent findings indicate that gestational and lactational supplementation of LY to sows increases the abundance of Ig-like domain-containing protein and complement proteins (Complement C8 alpha chain and C1q domain-containing proteins) in the colostrum and IgG heavy chain in milk (Fu et al., 2025). Similar findings also suggest that maternal dietary LY supplementation improves IgG concentrations in the colostrum and subsequently increases plasma IgG levels in piglets (Jang et al., 2013). Thus, these enhancements in colostrum and milk immunological quality may modulate the development and responsiveness of the offspring’s immune system (Reyes-Camacho et al., 2020).

The LPS challenge induced a decrease in villus height and crypt depth in the jejunum of piglets, indicating the potential intestinal injury following endotoxin exposure. This is consistent with previous findings that LPS induces morphological alterations in the digestive tract and increased mucosal permeability (Li et al., 2022a). The intestinal barrier is primarily composed of epithelial cells that are tightly interconnected by adherence and junction complexes, including E-cadherin, claudins, and occludin, to form a selectively permeable barrier that maintains intestinal integrity (Zhang et al., 2015). Reduced expression of these proteins may indicate a disruption of the intestinal barrier, contributing to increased intestinal permeability and facilitating the translocation of luminal antigens and bacteria into systemic circulation (Liu et al., 2023). In the current study, the effect of maternal LY supplementation on tight junction proteins was dependent on LPS challenge, as reflected in the different abundances of jejunal occludin and ileal claudin-4. In addition, piglets from LY-supplemented sows had a higher protein abundance of E-cadherin in the jejunal mucosa compared with piglets from CON sows, regardless of LPS challenge. This maternal effect suggests that LY supplementation may enhance epithelial cell adhesion and support mucosal integrity under both normal and challenged conditions. This pattern is consistent with our previous findings that gestational and lactation LY supplementation increases the abundance of junctional proteins such as E-cadherin, occludin, and claudin-3 in the small intestines of suckling and newly weaned piglets (Fu et al., 2024). Maternal nutrition modulates the maternal entero-mammary route and colostrum or milk composition, which may affect epithelial development and tight junction expression of offspring (Li et al., 2022b). In addition, our data indicate that maternal LY supplementation enriched bacterial taxa associated with SCFA production in piglets during suckling and early postweaning period (Y. Fu, unpublished data). The SCFAs, especially butyrate, strengthen the intestinal barrier by activating AMP-activated protein kinase, which promotes the assembly and reorganization of tight junction proteins (Peng et al., 2009). Future studies are needed to identify the specific yeast-derived components and metabolites that mediate these maternal programming effects on neonatal intestinal development and immune maturation, whether through modulation of placental nutrient and immune signaling or through direct transfer to the offspring via colostrum and milk.

Overall, supplementing sow diets with LY partially inhibited the inflammatory response to LPS challenge in the liver, lymph node, and intestinal mucosa of newly weaned piglets, supporting the possibility that LY exerts immunomodulatory effects through maternal-neonatal transfer. However, there is a nuanced effect of LY in the modulation of the inflammatory response to LPS that is tissue specific, illustrating the complexity of whole-body immune regulation.

## Conclusion

In conclusion, although the LPS challenge induced an inflammatory response characterized by elevated rectal temperature, intestinal barrier disruption, oxidative stress, and tissue-specific inflammatory response, gestational and lactational dietary supplementation with LY partially alleviated these adverse effects. Key points of LY effects included suppression of TLR4-mediated pro-inflammatory signaling pathways in the liver and lymph nodes, reducing local inflammation, and improving adherens junction protein expression in the intestinal mucosa. These findings suggest that maternal dietary live yeast supplementation could enhance the immune resilience of piglets, offering a promising nutritional strategy to mitigate weaning stress and inflammation during the early postweaning period.

## Supplementary Material

skaf435_Supplementary_Data

## Abbreviations

CAT: catalase
CD: crypt depth
CS: control + saline
CLPS: control + lipopolysaccharide
GAPDH: glyceraldehyde 3-phosphate dehydrogenase
GPX1: glutathione peroxidase 1
IKBA: inhibitor of nuclear factor kappa B alpha
IL: interleukin
LY: live yeast
LPS: lipopolysaccharide
MyD88: myeloid differentiation factor 88
NF-κB: nuclear factor kappa B
RT-qPCR: reverse transcription quantitative polymerase chain reaction
SCFAs: short-chain fatty acids
SOD: superoxide dismutase
TNF: tumor necrosis factor
TLR: toll-like receptor
VH: villus height
YS: yeast + saline
YLPS: yeast + lipopolysaccharide
