# Supplementary material for: Gestational and lactational dietary supplementation with live yeast partially attenuates inflammatory responses to lipopolysaccharide challenge in newly weaned piglets
Source: J Anim Sci. 2025 Dec 15;104:skaf435. doi: 10.1093/jas/skaf435 (PMC12863951; doi:10.1093/jas/skaf435)
Supplement: skaf435_Supplementary_Data [file skaf435_supplementary_data.zip › Supplementary_File.docx]

**Supplementary File**

Figure S1. Effects of maternal dietary live yeast supplementation on tight junction protein expression in the (A) jejunal and (B) ileal mucosa of newly weaned piglets in response to a lipopolysaccharide challenge (n = 8). Abbreviations: CS, control + saline; YS, yeast + saline; CLPS, control + lipopolysaccharide; YLPS, yeast + lipopolysaccharide.


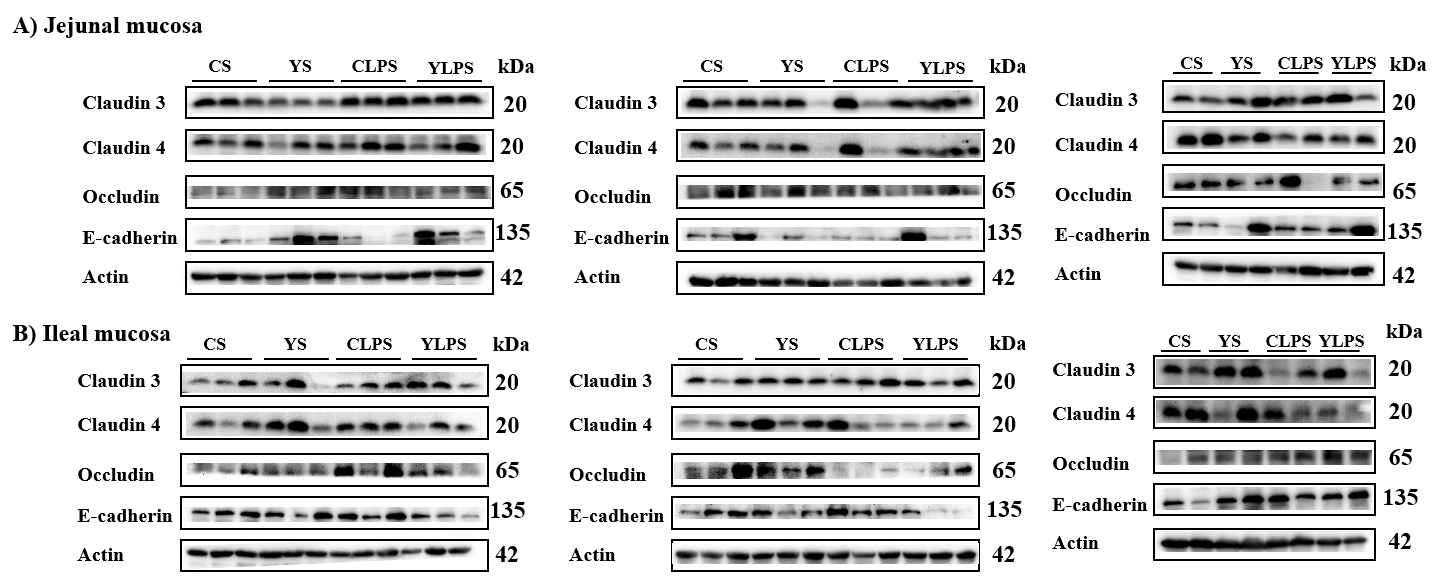


Table S1. Sequences of primers used for quantitative real-time PCR.

| Gene | Sequence (5ʹ → 3ʹ) | Accession no. | Product size |
| --- | --- | --- | --- |
| IL-6 | F-TCTGGGTTCAATCAGGAGACCTGC R-TGCACGGCCTCGACATTTCCC | NM_214399.1 | 122 |
| IL-10 | F-TGCCCAGCTCAGCACTGCTC R-CACTGGGCCGAAGGCAGCTC | NM_214041.1 | 140 |
| TNF-α | F-CGTCGCCCACGTTGTAGCCAAT R-GCCCATCTGTCGGCACCACC | NM_214022.1 | 128 |
| *SOD1* | F-CAGGGCACCATCTACTTCGAG R-ATGATCACCTTCAGCCAGTCC | NM_001190422.1 | 84 |
| CAT | F-CCTGCAACGTTCTGTAAGGC R-GCTTCATCTGGTCACTGGCT | NM_214301.2 | 72 |
| GPX1 | F-TACAGCCGTCGCTTTCTGAC R-CACTCTAGGCACTGCTAGGC | NM_214201.1 | 82 |
| TLR4 | F-GACAGCAATAGCTTCTCCAGC  R-GCCCTGATATGCATCATCGTC | NM_001113039.2 | 93 |
| MYD88 | F-CCATTCGAGATGACCCCCTG  R-TGCACAAACTGGGTATCGCT | NM_001099923.1 | 85 |
| IKBA | F-AGCGAGGATGAGGAGAGCTA  R-CCAAGCACGCAGTCGTCATA | [NM_001005150.1](https://www.ncbi.nlm.nih.gov/entrez/viewer.fcgi?db=nucleotide&id=52346211) | 77 |
| *NF-κB* | F-TCAACAAAGTGCCCCACGAT  R-GGAAACTGCCAGCAGGCTAA | NM_001048232.1 | 72 |
| GAPDH | F-GTTTGTGATGGGCGTGAAC R-ATGGACCGTGGTCATGAGT | NM_001206359.1 | 148 |

Abbreviations: CAT, catalase; GAPDH, glyceraldehyde 3-phosphate dehydrogenase; GPX1, glutathione peroxidase 1; IKBA, inhibitor of nuclear factor kappa B alpha; IL, interleukin; MyD88, myeloid differentiation factor 88; NF-κB, nuclear factor kappa B; SOD, superoxide dismutase; TNF, tumor necrosis factor; TLR, toll-like receptor.

Table S2. Effects of maternal live yeast supplementation on mRNA expression of inflammatory- and antioxidant-related markers in the muscle of newly weaned piglets in response to lipopolysaccharide challenge.

| Maternal diet | CON | | LY | | *SEM* | *P*-value | | |
| --- | --- | --- | --- | --- | --- | --- | --- | --- |
|  | Saline | LPS | Saline | LPS |  | Maternal diet | Challenge | Maternal diet x Challenge |
| *SOD1* | 1.00 | 0.77 | 0.80 | 0.78 | 0.10 | 0.31 | 0.20 | 0.29 |
| *GPX1* | 1.00 | 0.83 | 0.78 | 1.44 | 0.23 | 0.41 | 0.31 | 0.10 |
| *CAT* | 1.00 | 0.74 | 0.87 | 0.80 | 0.10 | 0.76 | 0.12 | 0.34 |
| *IL-6* | 1.00 | 1.22 | 0.73 | 1.06 | 0.15 | 0.18 | 0.09 | 0.73 |
| *IL-10* | 1.00 | 1.05 | 0.76 | 1.05 | 0.10 | 0.24 | 0.12 | 0.26 |
| *TNF-α* | 1.00 | 0.61 | 0.60 | 0.89 | 0.22 | 0.80 | 0.83 | 0.15 |

Abbreviations: CON, control; CAT, catalase; GPX1, glutathione peroxidase 1; LY, live yeast; SOD, superoxide dismutase; TNF, tumor necrosis factor; (n = 8).
